# Optogenetic activation of dopamine receptor D1 and D2 neurons in anterior cingulate cortex differentially modulates trigeminal neuropathic pain

**DOI:** 10.1007/s12035-020-02020-2

**Published:** 2020-07-11

**Authors:** Sufang Liu, Hui Shu, Joshua Crawford, Yajing Ma, Changsheng Li, Feng Tao

**Affiliations:** a Department of Biomedical Sciences, Texas A&M University College of Dentistry, Dallas, Texas, USA;; b Department of Physiology and Neurobiology, Zhengzhou University School of Medicine, Zhengzhou, Henan, China;; c Department of Anesthesiology, Zhengzhou University School of Medicine, Zhengzhou, Henan, China;; d Center for Craniofacial Research and Diagnosis, Texas A&M University College of Dentistry, Dallas, Texas, USA.

**Keywords:** Dopamine receptors, Anterior cingulate cortex, Trigeminal neuropathic pain, Optogenetic stimulation

## Abstract

**Background:**

Anterior cingulate cortex (ACC) is a critical brain center for chronic pain processing. Dopamine signaling in the brain has been demonstrated to contribute to descending pain modulation. However, the role of ACC dopamine receptors in chronic neuropathic pain remains unclear.

**Objective:**

In this study, we investigated the effect of optogenetic activation of ACC dopamine receptors D1- and D2-expressing neurons on trigeminal neuropathic pain.

**Methods:**

Chronic constriction injury of infraorbital nerve (CCI-ION) was carried out to induce trigeminal neuropathic pain in mice. We conducted optogenetic stimulation to specifically activate D1- and D2-expressing neurons in the ACC. Western blotting and immunofluorescence staining were used to examine ACC D1 and D2 expression and localization. The von Frey and real-time place preference tests were performed to measure evoked mechanical pain and nonreflexive emotional pain behaviors, respectively.

**Results:**

We observed that dopamine receptors D1 and D2 in the ACC are primarily expressed in excitatory neurons and that the D2 receptor is differentially regulated in the early and late phases of trigeminal neuropathic pain. Optogenetic activation of D1-expressing neurons in the ACC markedly exacerbates CCI-ION-induced trigeminal neuropathic pain in both early and late phases, but optogenetic activation of D2-expressing neurons in the ACC robustly ameliorates such pain in its late phase.

**Conclusion:**

Our results suggest that dopamine receptors D1 and D2 in the ACC play different roles in the modulation of trigeminal neuropathic pain.

## 1. INTRODUCTION

Dopamine signaling in the brain is involved in descending pain modulation. Previous studies have shown that the regulation of dopamine receptors can be used as a therapeutic approach for trigeminal neuralgia [1–4], which is characterized by chronic neuropathic pain in facial skin innervated by trigeminal nerve [5,6,3,7]. Anterior cingulate cortex (ACC) is a critical brain center for chronic pain processing, especially for its affective component [8–10]. Dopamine receptors in the ACC contribute to a number of neuropsychiatric and neurological disorders, including schizophrenia, depression, and pain [11,12,9,13–17]. However, it is unknown whether different dopamine receptors in the ACC differentially mediate pain modulation.

It has been demonstrated that dopamine receptors are metabotropic G protein-coupled receptors and they are divided into D1 subfamily (D1 and D5) and D2 subfamily (D2, D3, and D4). Our recent work revealed that activation of D1 and D2 receptors in the spinal trigeminal nucleus caudalis (Sp5C) oppositely modulates trigeminal neuropathic pain [18]. We further found that Sp5C D2, but not D1, mediates descending pain inhibition [18]. These results suggest that D1 and D2 receptors at the brainstem level (such as Sp5C) are differentially involved in the modulation of trigeminal neuropathic pain, but the role of these dopamine receptors at the cortical level in pain modulation is still unclear.

In the present study, we examine changes in expression of D1 and D2 receptors in the ACC after chronic construction nerve injury of the infraorbital nerve (CCI-ION), and we employ optogenetic stimulation to manipulate D1 or D2 receptor activity and investigate the effect of optogenetic activation of ACC D1- and D2-expressing neurons on CCI-ION-induced trigeminal neuropathic pain in both early and late phases.

## 2. MATERIALS AND METHODS

### 2.1. Animals

C57BL/6 wild-type male mice (8–10 weeks) from the Jackson Laboratory, D1-Cre and D2-Cre transgenic male mice (8–10 weeks) on a C57BL/6 background [19–21] from GENSAT, and Ai14 mice (B6.Cg-Gt(ROSA)26Sortm14(CAG-tdTomato)Hze/J) on a C57BL/6 background from the Jackson Laboratory were used. The mice were housed under standard conditions with a 12 h light-dark cycle, and water and food pellets were available *ad libitum*. Animal groups were assigned randomly. Mice were acclimated for 60 minutes prior to behavioral testing. All behavioral tests were conducted by an investigator who was blinded to the treatment group. All experiments comply with the guidelines of “Animal Research: Reporting of In Vivo Experiments (ARRIVE)” [22] and were conducted in accordance with the ethical guidelines of the National Institutes of Health for the care and use of laboratory animals. All efforts are made to minimize the suffering of animals and reduce the number of animals used. All experiments were approved by the Animal Care and Use Committee at the Texas A&M University College of Dentistry.

### 2.2. Trigeminal neuropathic pain mouse model

We carried out CCI-ION to induce trigeminal neuropathic pain in mice as described previously [23,18]. The CCI-ION has been considered as a classical neuropathic pain model [24] as infraorbital nerve (ION) does not include any autonomic components. In brief, the mice were anesthetized with pentobarbital sodium (50 mg/kg, i.p.) and the ION was exposed through a 2 mm incision in the palatalbuccal mucosa using sterile technic. We loosely tied the ION with two 4–0 chromic gut ligatures. The incision was closed with tissue adhesive. Control mice received only nerve exposure without ligation.

### 2.3. Optogenetic activation of dopamine receptors D1- and D2-expressing neurons

D1-Cre and D2-Cre mice were injected with the following Cre-inducible viruses into unilateral ACC (contralateral to the CCI-ION side) according to predetermined coordinates (AP, 1.34 mm; ML, 0.3 mm; DV, 1.25 mm) [25,26]: 1) a control virus (AAV5-EF1α-DIO-EYFP); 2)a virus expressing channelrhodopsin-2 (ChR2) for neuronal excitation (AAV5-EF1α-DIO-ChR2 (E123A)-EYFP-WPRE). Each virus (0.5 μl) was injected into ACC at 0.1 μl/min for optogenetic stimulation. After injection, the needle (33 gauge, Hamilton) was remained in place for 5 min before being retracted from ACC. Next, an optical fiber with a 1.25 mm ferrule cannula was implanted 0.3 mm above the virus injection site through the same hole after syringe removal [27]. The diode-pumped solid-state laser (473 nm blue light) produced from an OEM Laser System (Midvale, Utah) was used for optogenetic activation of ACC D1/2-expressing neurons. The light stimulation was performed at 20 Hz and the light intensity was 3–5 mW. After experiments, the location of infusion and light stimulation site was confirmed histologically.

### 2.4. Orofacial mechanical pain test with von Frey filaments

We used a series of calibrated von Frey filaments (0.07, 0.16, 0.25, 0.40, 0.60, 1.2, and 2.0 g) to measure orofacial mechanical pain. Each mouse was placed in a 10-cm long plexiglass viewing cylinder and allowed to poke out their heads and forepaws, but the mouse cannot turn around in the cylinder. After 60 min of acclimation, the von Frey filament was applied to the trigeminal nerve V2 branch-innervated facial skin. A positive response was defined as a sharp withdrawal of the head upon stimulation. Each filament was applied five times to the V2-innervated skin area for 1 s at 10 s intervals, starting from the lowest force of the filament (0.07 g) and continuing in ascending order. The head withdrawal threshold was calculated as the force at which the positive response occurred in three of five stimuli, which is an indicator for sensory component of pain in the CCI-ION model.

### 2.5. Real-time place preference (RTPP) test

According to a previous study [28], the RTPP test was performed in a standard three-chamber device with two large rooms and a buffer chamber. The two large rooms have different visual appearances, one with white stripes on the black walls and the other with only black walls. Mouse activity in each room was automatically recorded by a high-speed video camera and analyzed using the ANY-maze software (Stoelting, IL). Mice were first measured for 15 minutes as their baseline of the RTPP test. Next, optogenetic stimulation was performed when the mice entered the designated stimulating room, while the mice did not receive stimulation in another room and buffer chamber. The total test duration is 15 minutes. The affective component of trigeminal neuropathic pain induced by CCI-ION was determined by analyzing the time spent in the designated stimulating room during optogenetic stimulation.

### 2.6. Western blotting

Mice were sacrificed under isoflurane anesthesia and the ACC tissues were harvested on ice. The primary antibodies against D1 (Cat. # MAB5290, Millipore, 1:330) and D2 (Cat. # sc-5303, Santa Cruz Biotechnology, 1:300) were used to assess the expression level of D1 and D2 in the ACC. β-actin (Cat # A5316, Sigma) served as a loading control in all Western blotting experiments. The intensities of bands in the Western blotting were quantified with densitometry. The intensity values of D1/2 bands were normalized to β-actin and expressed as a ratio of D1/ β-actin or D2/ β-actin.

### 2.7. Immunofluorescence staining

To determine the expression of dopamine receptors D1 and D2 in the ACC, *D1-Cre:Ai14* and *D2-Cre:Ai14* mouse lines were generated by crossing a homozygous D1-Cre or D2-Cre mouse with an Ai14 mouse, and the resulting heterozygous *D1-Cre:Ai14* and *D2-Cre:Ai14* offspring mice were used for immunofluorescence staining, in which D1- or D2-expressing neurons are labeled with red tdTomato. The offspring mice were perfused transcardially with saline followed by 4% paraformaldehyde (PFA). Following the perfusion, ACC-containing brain tissues were cut at 20 μm with a cryostat (CM1950, Leica, Chicago, IL). Free-floating slices were blocked in a 5% normal goat serum for 1 h followed by incubation with primary antibodies overnight at 4 °C. The primary antibodies include anti-NeuN (Abcam, Cat. # ab104224, 1:500), anti-glial fibrillary acidic protein (GFAP, Millipore, Cat. # AB5541, 1:800), anti-ionized calcium binding adapter molecule 1(Iba1, Wako Chemicals, Cat. # 019–19741, 1:400), anti-T-cell leukemia 3 (TLX3, ThermoFisher, Cat. # PA534555, 1:200) and anti-paired box 2 (PAX2, ThermoFisher, Cat. # 71–6000, 1:500). Next, the slices were washed and placed in a corresponding secondary antibody conjugated to Alexa Fluor 488 for 1 h at room temperature. Images were taken using a Leica fluorescence microscope (DMi8, Leica).

### 2.8. Statistical analysis

Data are expressed as the mean ± S.E.M. Sample size calculations were performed using the power analysis program G*Power 3.1 [29]. Statistical analyses were conducted with GraphPad Prism 8 software. Unpaired *t*-test was used to analyze Western blot data and one-way analysis of variance (ANOVA) followed by the Tukey *post-hoc* test was used to analyze behavioral data. The level of statistical significance was set at *P* < 0.05.

## 3. RESULTS

### 3.1. Dopamine receptors D1 and D2 are primarily expressed in excitatory neurons of the ACC

**Fig. 1:**
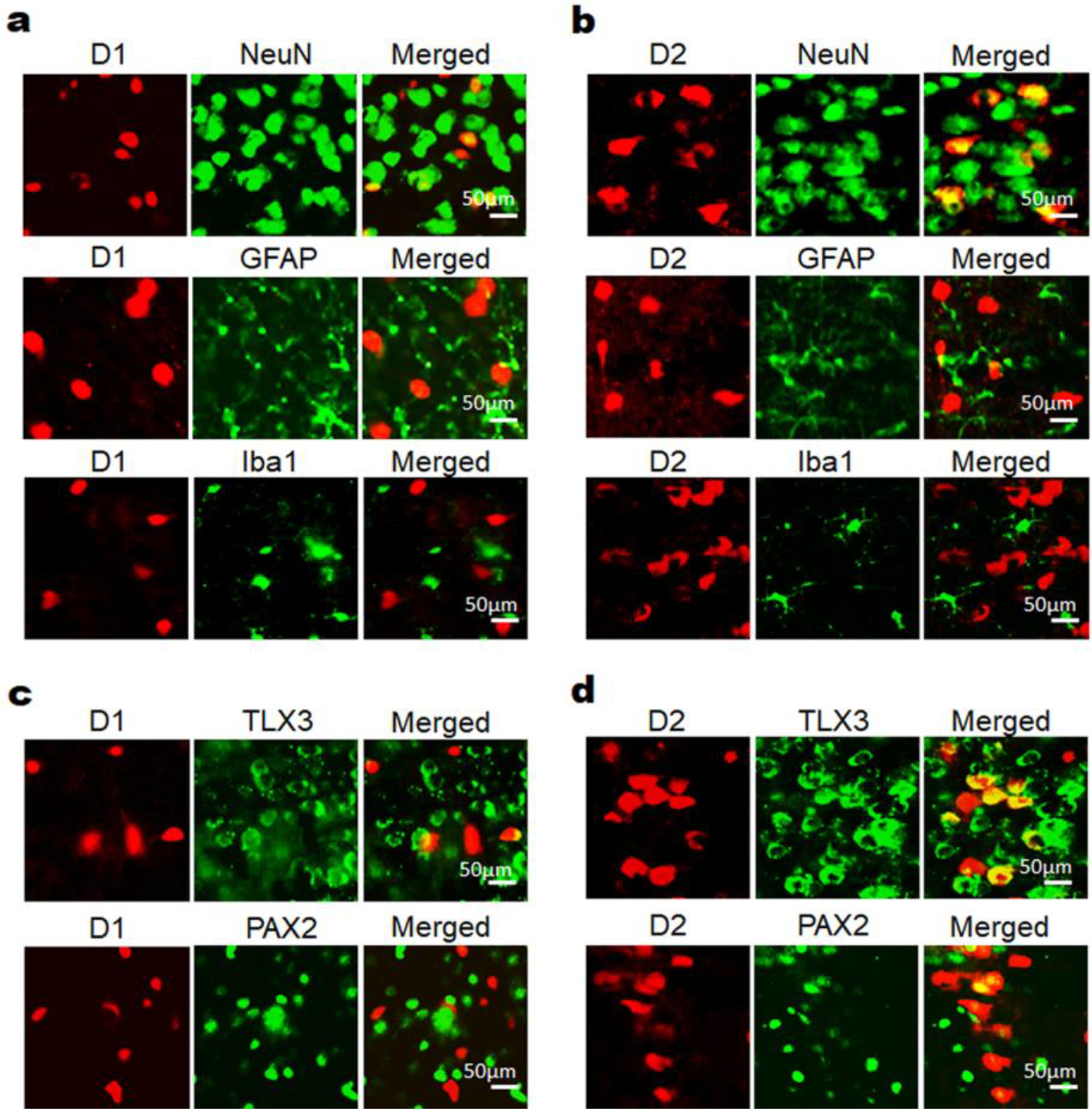
Dopamine receptors D1 and D2 are primarily expressed in excitatory neurons of the ACC. *D1-Cre:Ai14* and *D2-Cre:Ai14* mouse lines were generated to label D1 and D2, respectively, with red fluorescent protein tdTomato. The offspring mice were used for immunofluorescence staining with different cell markers. (a) Immunofluorescence staining with NeuN, GFAP andIba1 using ACC sections of *D1-Cre:Ai14* mice showed that tdTomato-labeled D1 in the ACC was exclusively expressed in NeuN-positive neurons, but not GFAP-positive or Iba1-positive glial cells. (b) Immunofluorescence staining with NeuN, GFAP and Iba1 using ACC sections of *D2-Cre:Ai14* mice showed that tdTomato-labeled D2 in the ACC was also exclusively expressed in NeuN-positive neurons, but not GFAP-positive or Iba1-positive glial cells. (c) Immunofluorescence staining with TLX3 and PAX2 using ACC sections of *D1-Cre:Ai14* mice showed that tdTomato-labeled D1 in the ACC was mostly expressed in TLX3-positive excitatory neurons, but not in PAX2-positive inhibitory neurons. (d) Immunofluorescence staining with TLX3 and PAX2 using ACC sections of *D2-Cre:Ai14* mice showed that tdTomato-labeled D2 in the ACC was expressed in both TLX3-positive excitatory neurons and PAX2-positive inhibitory neurons, but the majority of D2 was co-labeled with TLX3. The immunofluorescence staining experiments were repeated three times to confirm the data shown in this figure. Scale bar, 50 μm.

To specifically localize D1- or D2-expressing neurons in the ACC, we generated *D1-Cre:Ai14* and *D2-Cre:Ai14* mouse lines by crossing homozygous D1-Cre or D2-Cre mice with Ai14 mice. Ai14 is a Cre reporter allele designed to have a loxP-flanked STOP cassette preventing transcription of a CAG promoter-driven red fluorescent protein tdTomato, and the resulting heterozygous *D1-Cre:Ai14* and *D2-Cre:Ai14* mice expressed robust tdTomato due to Cre-mediated recombination. These mice were used for immunofluorescence staining with different cell markers, including NeuN (a marker for neurons), GFAP (a marker for astrocytes), and Iba1 (a marker for microglia). We observed that tdTomato-labeled D1 in the ACC of *D1-Cre:Ai14* mice was exclusively expressed in NeuN-positive neurons, but not GFAP-positive or Iba1-positive glial cells (Fig. 1a). Similarly, tdTomato-labeled D2 in the ACC of *D2-Cre:Ai14* mice was also exclusively expressed in NeuN-positive neurons, but not GFAP-positive or Iba1-positive glial cells (Fig. 1b).

Moreover, we further used the offspring mice to perform immunofluorescence staining with an excitatory neuron marker TLX3 [30] and an inhibitory neuron marker PAX2 [31,32]. We observed that tdTomato-labeled D1 in the ACC of *D1-Cre:Ai14* mice was mostly expressed in TLX3-positive excitatory neurons, but not in PAX2-positive inhibitory neurons (Fig. 1c), and that tdTomato-labeled D2 in the ACC of *D2-Cre:Ai14* mice was expressed in both TLX3-positive excitatory neurons and PAX2-positive inhibitory neurons, but the majority of D2 was co-labeled with TLX3 (Fig. 1d).

### 3.2. CCI-ION differentially regulates the expression of D1 and D2 in the ACC

**Fig. 2:**
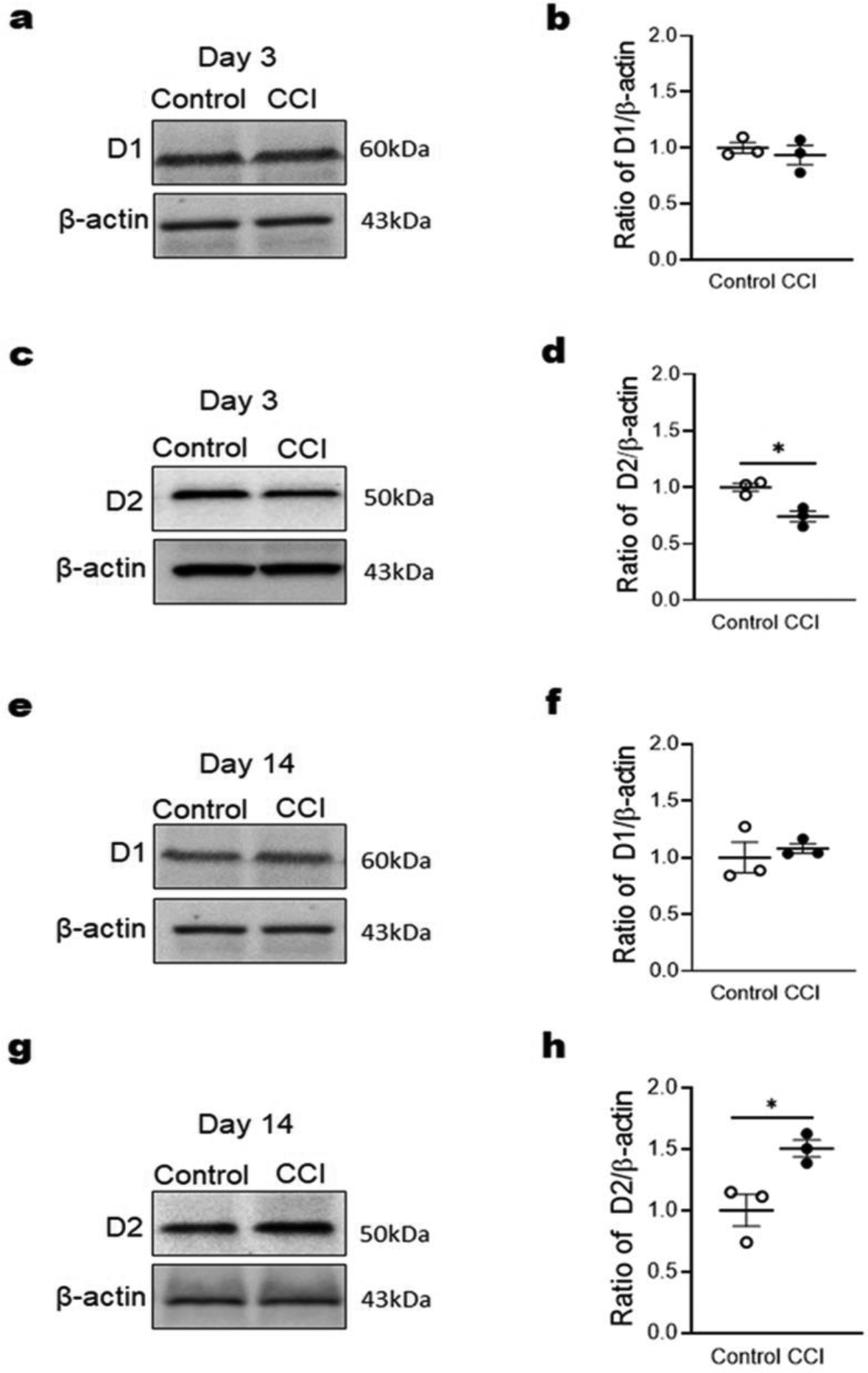
Dopamine receptors D1 and D2 in the ACC are differentially regulated by CCI-ION. Western blotting was performed to examine change in the expression of these receptors in the ACC on day 3 and day 14 after CCI-ION. (a and b) ACC D1 expression had no significant alteration on day 3 after CCI-ION. (c and d) ACC D2 expression was significant decreased on day 3 after CCI-ION. (e and f) ACC D1 expression had no significant alteration on day 14 after CCI-ION. (g and h) ACC D2 expression was significant increased on day 14 after CCI-ION. n = 3 per group. *P < 0.05 *vs.* the sham control group.

To reveal whether D1 and D2 receptors in the ACC are regulated during trigeminal neuropathic pain, we performed Western blotting to examine changes in the expression of these receptors in the ACC on day 3 and day 14 after CCI-ION. Our results showed that in the early phase of the neuropathic pain (Day 3 post-surgery), the expression of D1 in the ACC had no significant change (Fig. 2a and b), but ACC D2 level was significantly decreased compared to the sham control group (Fig. 2c and d). In the late phase of the neuropathic pain (Day 14 post-surgery), the expression of D1 in the ACC still had no significant change (Fig. 2e and f), but ACC D2 level was significantly increased compared to the sham control group (Fig. 2g and h).

### 3.3. Optogenetic activation of DA receptors D1- and D2-expressing neurons in the ACC differentially modulates trigeminal neuropathic pain

**Fig. 3:**
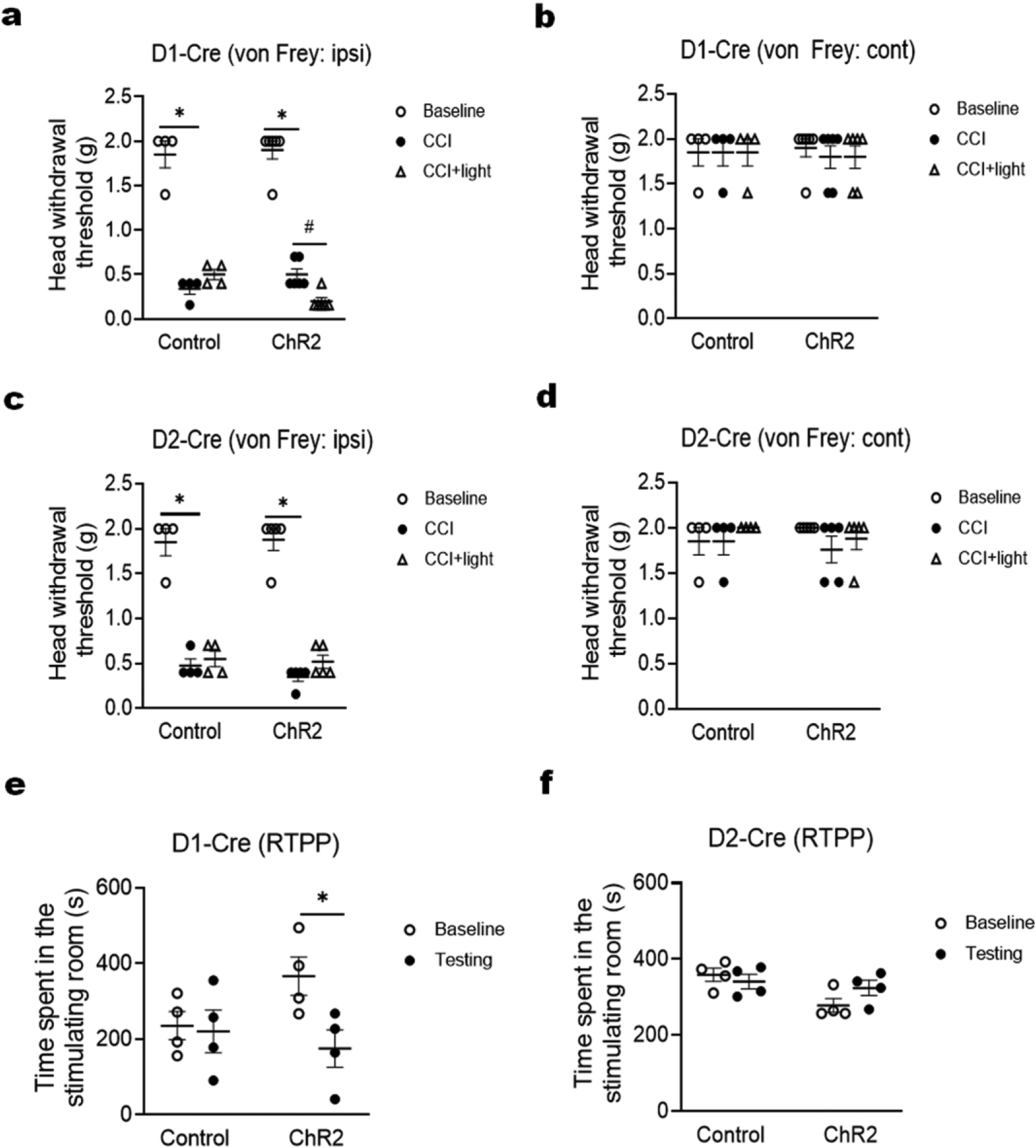
Optogenetic activation of ACC dopamine receptor D1-expressing neurons, but not D2-expressing neurons, increases trigeminal neuropathic pain on day 3 after CCI-ION. (a and c) In both D1-Cre (a) and D2-Cre (c) mice, the CCI-ION significantly decreased head withdrawal threshold in the ipsilateral side; however, optogenetic activation of ACC D1 neurons (a), but not D2 neurons (c), further decreased the head withdrawal threshold. (b and d) In both D1-Cre (b) and D2-Cre (d) mice, the CCI-ION and optogenetic stimulation had no effect on head withdrawal threshold in the contralateral side. (e and f) In the real-time place preference (RTPP) test, optogenetic activation of ACC D1 neurons (e), but not D2 neurons (f), significantly decreased the time spent in stimulating room. n = 6 per group. *P < 0.05 *vs.* the corresponding Baseline values; ^#^*P* < 0.05 *vs.* the corresponding CCI group.

To determine the role of ACC DA receptors D1 and D2 in trigeminal neuropathic pain, we applied optogenetic stimulation to specifically manipulate these receptors. In the early phase of the CCI-ION-induced neuropathic pain (Day 3 post-surgery), we observed that CCI-ION significantly decreased head withdrawal threshold on the ipsilateral side. Interestingly, optogenetic activation of ACC D1-expressing neurons, but not D2-expressing neurons, further decreased the head withdrawal threshold (Fig. 3a and c). Meanwhile, CCI-ION and optogenetic stimulation had no effect on head withdrawal threshold on the contralateral side (Fig. 3b and d). Using the RTPP test, we observed that optogenetic activation of ACC D1-expressing neurons, but not D2-expressing neurons, significantly decreased the time spent in the stimulating room (Fig. 3e and f). These results indicate that CCI-ION-induced neuropathic pain (both sensory and affective component) in the early phase is enhanced by activation of ACC D1-expressing neurons.

**Fig. 4:**
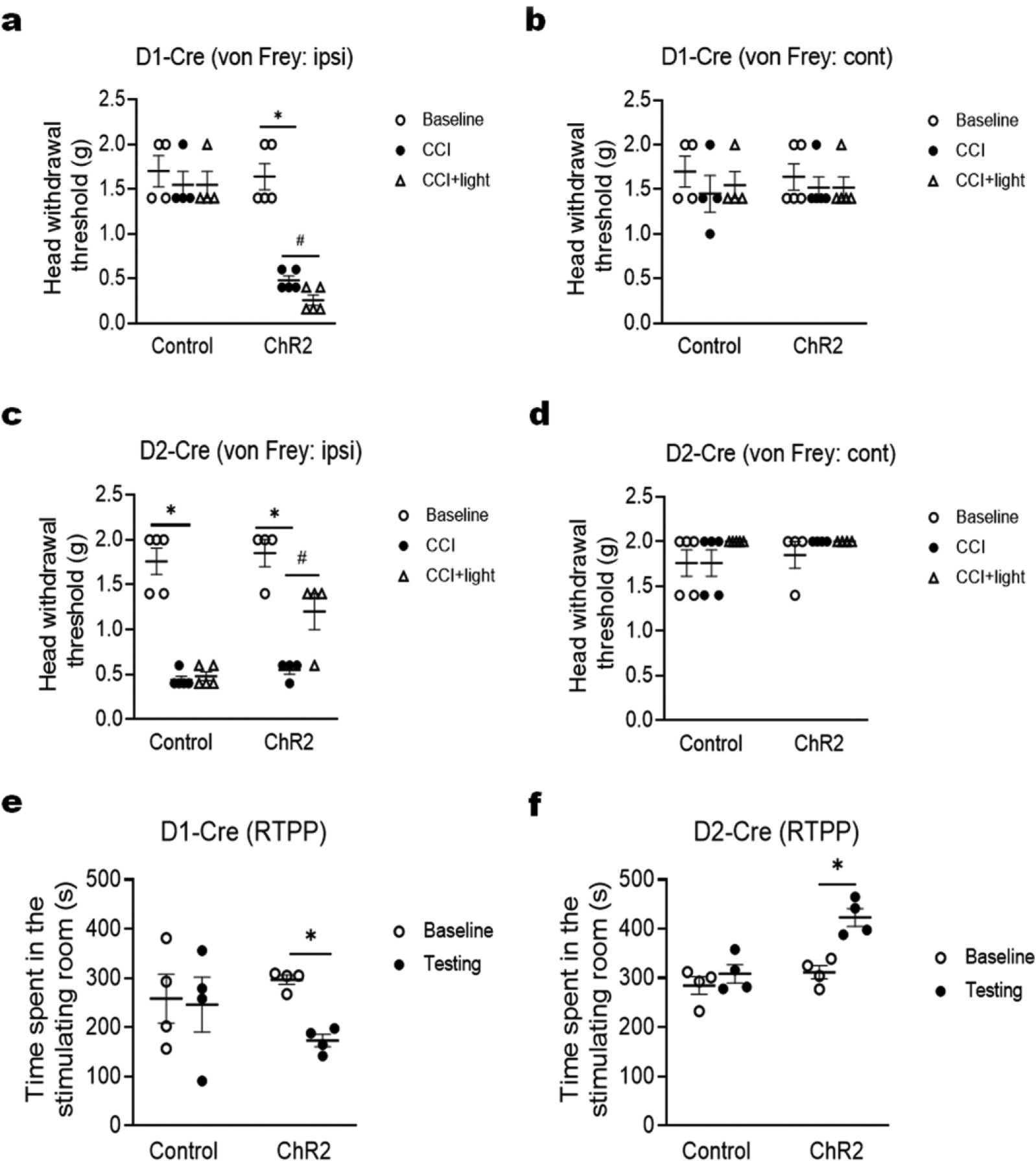
Optogenetic activation of ACC dopamine receptor D1-expressing neurons and D2-expressing neurons oppositely modulates trigeminal neuropathic pain on day 14 after CCI-ION. (a and c) In D1-Cre mice (a), optogenetic activation of ACC D1 neurons further decreased head withdrawal threshold in the ipsilateral side; in D2-Cre mice (c), optogenetic activation of ACC D2 neurons reversely increased head withdrawal threshold in the ipsilateral side. (b and d) In both D1-Cre (b) and D2-Cre (d) mice, the CCI-ION and optogenetic stimulation had no effect on head withdrawal threshold in the contralateral side. (e and f) In the real-time place preference (RTPP) test, optogenetic activation of ACC D1 neurons (e) significantly decreased the time spent in stimulating room, but optogenetic activation of ACC D1 neurons (f) significantly increased the time spent in stimulating room. n = 6 per group. *P < 0.05 *vs.* the corresponding Baseline values; ^#^*P* < 0.05 *vs.* the corresponding CCI group.

In the late phase of CCI-ION-induced neuropathic pain (Day 14 post-surgery), we observed that CCI-ION continually decreased head withdrawal threshold on the ipsilateral side. Strikingly, optogenetic activation of ACC D1- and D2-expressing neurons oppositely affected the head withdrawal threshold: D1 neuron activation decreased it, but D2 neuron activation increased it (Fig. 4a and c). Similar to the early phase, CCI-ION and optogenetic stimulation had no effect on head withdrawal threshold during the late phase on the contralateral side (Fig. 4b and d). Using the RTPP test, we verified that optogenetic activation of ACC D1- and D2-expressing neurons oppositely affected the time spent in the stimulating room (Fig. 4e and f). These results indicate that CCI-ION-induced neuropathic pain (both sensory and affective components) in the late phase is enhanced by activation of ACC D1-expressing neurons, but inhibited by activation of ACC D2-expressing neurons.

## 4. DISCUSSION

In the present study, we observed that dopamine receptors D1 and D2 in the ACC are primarily expressed in excitatory neurons and that the D2 receptor is differentially regulated in the early and late phases of trigeminal neuropathic pain. Optogenetic activation of D1-expressing neurons in the ACC markedly exacerbates CCI-ION-induced trigeminal neuropathic pain in both early and late phases, but optogenetic activation of D2-expressing neurons in the ACC robustly ameliorates such pain in its late phase.

To date the expression of dopamine receptors in the ACC remains elusive. A previous study showed that dopamine receptors D1 and D2 mRNAs are expressed in the ACC of rats [9]. To better observe the expression of D1 and D2 receptors in the ACC, we crossed homozygous D1-Cre or D2-Cre mice with Ai14 mice to generate *D1-Cre:Ai14* and *D2-Cre:Ai14* mouse lines. Because the resulting offspring mice express robust tdTomato in D1 or D2 neurons due to Cre-mediated recombination, we can clearly observe the expression of these receptors in the ACC and other brain structures. Moreover, by combining with immunofluorescence staining, we revealed that ACC D1 and D2 receptors are primarily expressed in excitatory neurons. In contrast, D1 and D2 receptors are expressed in both excitatory and inhibitory neurons of the Sp5C as shown in our recent work [18]. Thus, the expression of these receptors at the cortical level is different from that at the brainstem level, which could underlie their function in the modulation of trigeminal neuropathic pain.

To define the involvement of ACC D1 and D2 receptors in trigeminal neuropathic pain, we examine changes in the expression of these receptors in the ACC after CCI-ION. Interestingly, we found that D2 receptor in the ACC is downregulated in the early phase of CCI-ION-induced trigeminal neuropathic pain (Day 3 post-surgery), but upregulated in the late phase of the neuropathic pain (Day 14 post-surgery). On the other hand, D1 receptor in the ACC has no significant change in both early and late phases of such pain. These results suggest that altered expression of D2 receptor in the ACC may contribute to the modulatory effect of dopamine signaling on chronic neuropathic pain. Since D1 and D2 receptors are rarely co-expressed in the same cells [33,34], we can manipulate them separately for pain treatment.

To further determine the role of ACC D1 and D2 receptors in trigeminal neuropathic pain, we applied optogenetic stimulation in our study. Optogenetic manipulation can produce cell-specific neuromodulation. Using optogenetic stimulation in D1-Cre and D2-Cre mice, we are able to conduct highly precise spatial and temporal control of D1 and D2 neurons in the ACC. Our data showed that optogenetic activation of ACC D1 and D2 neurons oppositely modulates CCI-ION-induced trigeminal neuropathic pain in its late phase. This finding is consistent with our recent work [18], in which we manipulated D1 and D2 neurons in the Sp5C and observed opposite pain modulatory effects. In this study, we used two pain behavioral tests (von Frey and RTPP) to measure different components of neuropathic pain: the von Frey test for assessing sensory component of pain; the RTPP test for assessing affective component of pain. Thus, our data provide comprehensive evidence to demonstrate that dopamine receptors D1 and D2 in the ACC are differentially involved in the modulation of trigeminal neuropathic pain.

Although D1 and D2, the most abundant dopamine receptors in the brain [35], are critically involved in the modulation of neuropathic pain, other dopamine receptor subunits (such as D5) have been reported to play an important role in pathological pain plasticity and chronic pain development [36,37]. Understanding the molecular mechanisms by which different dopamine receptors differentially mediate pain modulation will potentially help us identify new therapeutic targets for pain treatment. In addition, an interaction between dopaminergic and glutamatergic systems in the ACC has been suggested as a mechanism underlying development and maintenance of long-term nociception [38]. Thus, investigating potential involvement of ACC dopamine receptors in this interaction will explore a novel role for these receptors in the pathogenesis of chronic pain.

## 5. CONCLUSION

Our study reveals the expression of dopamine receptors D1 and D2 in the ACC and their regulation by CCI-ION. We further show that optogenetic activation of ACC D1 and D2 neurons differentially affects CCI-ION-induced trigeminal neuropathic pain. These results suggest that dopamine receptors D1 and D2 in the ACC play different roles in the modulation of trigeminal neuropathic pain.

## ABBREVIATIONS

ACC: anterior cingulate cortex
CCI-ION: chronic constriction injury of infraorbital nerve
ChR2: channelrhodopsin 2
GFAP: glial fibrillary acidic protein
Iba1: ionized calcium binding adapter molecule 1
NeuN: neuronal nuclei
PAX2: paired box 2
RTPP: real-time place preference
Sp5C: spinal trigeminal nucleus caudalis
TLX3: T-cell leukemia 3

## References

[1] Lechin F, van der Dijs B, Lechin ME, Amat J, Lechin AE, Cabrera A, Gomez F, Acosta E, Arocha L, Villa S, (1989) Pimozide therapy for trigeminal neuralgia. Arch Neurol 46 (9):960–963. doi:10.1001/archneur.1989.00520450030015 2673161

[2] Zhang J, Yang M, Zhou M, He L, Chen N, Zakrzewska JM (2013) Non-antiepileptic drugs for trigeminal neuralgia. Cochrane Database Syst Rev (12):CD004029. doi:10.1002/14651858.CD004029.pub4 PMC11800189 24297506

[3] Dosenovic S, Kadic AJ, Miljanovic M, Biocic M, Boric K, Cavar M, Markovina N, Vucic K, Puljak L (2017) Interventions for Neuropathic Pain: An Overview of Systematic Reviews. Anesthesia and Analgesia 125 (2):643–652. doi:10.1213/Ane.0000000000001998 28731977

[4] Al-Quliti KW (2015) Update on neuropathic pain treatment for trigeminal neuralgia. The pharmacological and surgical options. Neurosciences (Riyadh) 20 (2):107–114. doi:10.17712/nsj.2015.2.20140501 25864062 PMC4727618

[5] Jones MR, Urits I, Ehrhardt KP, Cefalu JN, Kendrick JB, Park DJ, Cornett EM, Kaye AD, Viswanath O (2019) A Comprehensive Review of Trigeminal Neuralgia. Curr Pain Headache Rep 23 (10):74. doi:10.1007/s11916-019-0810-0 31388843

[6] Kumar S, Rastogi S, Kumar S, Mahendra P, Bansal M, Chandra L (2013) Pain in trigeminal neuralgia: neurophysiology and measurement: a comprehensive review. J Med Life 6 (4):383–388 24701256 PMC3973876

[7] Sabalys G, Juodzbalys G, Wang HL (2013) Aetiology and pathogenesis of trigeminal neuralgia: a comprehensive review. J Oral Maxillofac Res 3 (4):e2. doi:10.5037/jomr.2012.3402 24422020 PMC3886096

[8] Lu JS, Chen QY, Zhou S, Inokuchi K, Zhuo M (2018) Dual roles of anterior cingulate cortex neurons in pain and pleasure in adult mice. Mol Brain 11 (1):72. doi:10.1186/s13041-018-0416-1 30514335 PMC6280384

[9] Ortega-Legaspi JM, de Gortari P, Garduno-Gutierrez R, Amaya MI, Leon-Olea M, Coffeen U, Pellicer F (2011) Expression of the dopaminergic D1 and D2 receptors in the anterior cingulate cortex in a model of neuropathic pain. Mol Pain 7:97. doi:10.1186/1744-8069-7-97 22171983 PMC3286425

[10] DaSilva AF, Nascimento TD, Jassar H, Heffernan J, Toback RL, Lucas S, DosSantos MF, Bellile EL, Boonstra PS, Taylor JMG, Casey KL, Koeppe RA, Smith YR, Zubieta JK (2017) Dopamine D2/D3 imbalance during migraine attack and allodynia in vivo. Neurology 88 (17):1634–1641. doi:10.1212/WNL.0000000000003861 28356463 PMC5405765

[11] Darvish-Ghane S, Yamanaka M, Zhuo M (2016) Dopaminergic Modulation of Excitatory Transmission in the Anterior Cingulate Cortex of Adult Mice. Mol Pain 12. doi:10.1177/1744806916648153 PMC4955973 27317578

[12] Tian Z, Yamanaka M, Bernabucci M, Zhao MG, Zhuo M (2017) Characterization of serotonin-induced inhibition of excitatory synaptic transmission in the anterior cingulate cortex. Mol Brain 10 (1):21. doi:10.1186/s13041-017-0303-1 28606116 PMC5468981

[13] Navratilova E, Xie JY, Meske D, Qu C, Morimura K, Okun A, Arakawa N, Ossipov M, Fields HL, Porreca F (2015) Endogenous opioid activity in the anterior cingulate cortex is required for relief of pain. J Neurosci 35 (18):7264–7271. doi:10.1523/JNEUROSCI.3862-14.2015 25948274 PMC4420787

[14] Drevets WC, Savitz J, Trimble M (2008) The subgenual anterior cingulate cortex in mood disorders. CNS Spectr 13 (8):663–681 18704022 10.1017/s1092852900013754 PMC2729429

[15] Lidow MS, Goldman-Rakic PS, Rakic P, Innis RB (1989) Dopamine D2 receptors in the cerebral cortex: distribution and pharmacological characterization with [3H]raclopride. Proc Natl Acad Sci U S A 86 (16):6412–6416. doi:10.1073/pnas.86.16.6412 2548214 PMC297850

[16] Suhara T, Okubo Y, Yasuno F, Sudo Y, Inoue M, Ichimiya T, Nakashima Y, Nakayama K, Tanada S, Suzuki K, Halldin C, Farde L (2002) Decreased dopamine D2 receptor binding in the anterior cingulate cortex in schizophrenia. Arch Gen Psychiatry 59 (1):25–30. doi:10.1001/archpsyc.59.1.25 11779278

[17] Wei L, Hu X, Yuan Y, Liu W, Chen H (2018) Abnormal ventral tegmental area-anterior cingulate cortex connectivity in Parkinson’s disease with depression. Behav Brain Res 347:132–139. doi:10.1016/j.bbr.2018.03.011 29530489

[18] Liu S, Tang Y, Shu H, Tatum D, Bai Q, Crawford J, Xing Y, Lobo MK, Bellinger L, Kramer P, Tao F (2019) Dopamine receptor D2, but not D1, mediates descending dopaminergic pathway-produced analgesic effect in a trigeminal neuropathic pain mouse model. Pain 160 (2):334–344. doi:10.1097/j.pain.0000000000001414 30325872 PMC6344251

[19] Gong S, Doughty M, Harbaugh CR, Cummins A, Hatten ME, Heintz N, Gerfen CR (2007) Targeting Cre recombinase to specific neuron populations with bacterial artificial chromosome constructs. J Neurosci 27 (37):9817–9823. doi:10.1523/JNEUROSCI.2707-07.2007 17855595 PMC6672645

[20] Gong S, Zheng C, Doughty ML, Losos K, Didkovsky N, Schambra UB, Nowak NJ, Joyner A, Leblanc G, Hatten ME, Heintz N (2003) A gene expression atlas of the central nervous system based on bacterial artificial chromosomes. Nature 425 (6961):917–925. doi:10.1038/nature02033 14586460

[21] Gerfen CR, Paletzki R, Heintz N (2013) GENSAT BAC cre-recombinase driver lines to study the functional organization of cerebral cortical and basal ganglia circuits. Neuron 80 (6):1368–1383. doi:10.1016/j.neuron.2013.10.016 24360541 PMC3872013

[22] Kilkenny C, Browne WJ, Cuthill IC, Emerson M, Altman DG (2010) Improving bioscience research reporting: the ARRIVE guidelines for reporting animal research. PLoS Biol 8 (6):e1000412. doi:10.1371/journal.pbio.1000412 20613859 PMC2893951

[23] Kim YS, Chu Y, Han L, Li M, Li Z, Lavinka PC, Sun S, Tang Z, Park K, Caterina MJ, Ren K, Dubner R, Wei F, Dong X (2014) Central terminal sensitization of TRPV1 by descending serotonergic facilitation modulates chronic pain. Neuron 81 (4):873–887. doi:10.1016/j.neuron.2013.12.011 24462040 PMC3943838

[24] Kernisant M, Gear RW, Jasmin L, Vit JP, Ohara PT (2008) Chronic constriction injury of the infraorbital nerve in the rat using modified syringe needle. J Neurosci Methods 172 (1):43–47. doi:10.1016/j.jneumeth.2008.04.013 18501433 PMC2497464

[25] Juarez-Salinas DL, Braz JM, Etlin A, Gee S, Sohal V, Basbaum AI (2019) GABAergic cell transplants in the anterior cingulate cortex reduce neuropathic pain aversiveness. Brain 142 (9):2655–2669. doi:10.1093/brain/awz203 31321411 PMC6752168

[26] White MG, Panicker M, Mu C, Carter AM, Roberts BM, Dharmasri PA, Mathur BN (2018) Anterior Cingulate Cortex Input to the Claustrum Is Required for Top-Down Action Control. Cell Rep 22 (1):84–95. doi:10.1016/j.celrep.2017.12.023 29298436 PMC5779631

[27] Kusumoto-Yoshida I, Liu H, Chen BT, Fontanini A, Bonci A (2015) Central role for the insular cortex in mediating conditioned responses to anticipatory cues. Proc Natl Acad Sci U S A 112 (4):1190–1195. doi:10.1073/pnas.1416573112 25583486 PMC4313852

[28] Ferenczi EA, Zalocusky KA, Liston C, Grosenick L, Warden MR, Amatya D, Katovich K, Mehta H, Patenaude B, Ramakrishnan C, Kalanithi P, Etkin A, Knutson B, Glover GH, Deisseroth K (2016) Prefrontal cortical regulation of brainwide circuit dynamics and reward-related behavior. Science 351 (6268):aac9698. doi:10.1126/science.aac9698 26722001 PMC4772156

[29] Faul F, Erdfelder E, Lang AG, Buchner A (2007) G*Power 3: a flexible statistical power analysis program for the social, behavioral, and biomedical sciences. Behav Res Methods 39 (2):175–191. doi:10.3758/bf03193146 17695343

[30] Shimomura A, Patel D, Wilson SM, Koehler KR, Khanna R, Hashino E (2015) Tlx3 Promotes Glutamatergic Neuronal Subtype Specification through Direct Interactions with the Chromatin Modifier CBP. Plos One 10 (8). doi:ARTN e0135060 10.1371/journal.pone.0135060 10.1371/journal.pone.0135060 PMC4530954 26258652

[31] Larsson M (2017) Pax2 is persistently expressed by GABAergic neurons throughout the adult rat dorsal horn. Neuroscience Letters 638:96–101. doi:10.1016/j.neulet.2016.12.015 27939388

[32] Maricich SM, Herrup K (1999) Pax-2 expression defines a subset of GABAergic interneurons and their precursors in the developing murine cerebellum. Journal of Neurobiology 41 (2):281–294. doi:Doi 10.1002/(Sici)1097-4695(19991105)41:2<281::Aid-Neu10>3.0.Co;2-5 10512984

[33] Baik JH (2013) Dopamine signaling in reward-related behaviors. Front Neural Circuits 7:152. doi:10.3389/fncir.2013.00152 24130517 PMC3795306

[34] Missale C, Nash SR, Robinson SW, Jaber M, Caron MG (1998) Dopamine receptors: from structure to function. Physiol Rev 78 (1):189–225. doi:10.1152/physrev.1998.78.1.189 9457173

[35] Klein MO, Battagello DS, Cardoso AR, Hauser DN, Bittencourt JC, Correa RG (2019) Dopamine: Functions, Signaling, and Association with Neurological Diseases. Cell Mol Neurobiol 39 (1):31–59. doi:10.1007/s10571-018-0632-3 30446950 PMC11469830

[36] Kim JY, Tillu DV, Quinn TL, Mejia GL, Shy A, Asiedu MN, Murad E, Schumann AP, Totsch SK, Sorge RE, Mantyh PW, Dussor G, Price TJ (2015) Spinal dopaminergic projections control the transition to pathological pain plasticity via a D1/D5-mediated mechanism. J Neurosci 35 (16):6307–6317. doi:10.1523/JNEUROSCI.3481-14.2015 25904784 PMC4405552

[37] Megat S, Shiers S, Moy JK, Barragan-Iglesias P, Pradhan G, Seal RP, Dussor G, Price TJ (2018) A Critical Role for Dopamine D5 Receptors in Pain Chronicity in Male Mice. J Neurosci 38 (2):379–397. doi:10.1523/JNEUROSCI.2110-17.2017 29167404 PMC5761615

[38] Lopez-Avila A, Coffeen U, Ortega-Legaspi JM, del Angel R, Pellicer F (2004) Dopamine and NMDA systems modulate long-term nociception in the rat anterior cingulate cortex. Pain 111 (1–2):136–143. doi:10.1016/j.pain.2004.06.010 15327817

